# Supplementary material for: Rdh54/Tid1 inhibits Rad51-Rad54-mediated D-loop formation and limits D-loop length
Source: eLife. 2020 Nov 13;9:e59112. doi: 10.7554/eLife.59112 (PMC7695457; doi:10.7554/eLife.59112)
Supplement: Supplementary file 1. — 1 W303 strain background. 2 S288c strain background. 3 Obtained from Mehta et al., 2017. [file elife-59112-supp1.docx]

**Supplementary File 1: *Saccharomyces cerevisiae* strains used in this work.**

|  |  |
| --- | --- |
| Name | **Genotype** |
| WDHY5509^1^ | *MATa-inc, ura3::LY-HOcs, lys2::LYover0, trp1::GAL-HO-hphMX, his3D200, can1-100, leu2-3,112, ade2-1, RAD5* |
| WDHY5511^1^ | *MATα-inc, ura3::LY-HOcs, lys2::LYover0, trp1::GAL-HO-hphMX, his3D200, can1-100, leu2-3,112, ade2-1, RAD5* |
| WDHY5358^1^ | *MATa-inc, ura3::LY-HOcs, lys2::LYover0, trp1::GAL-HO hphMX, his3, leu2-3,112, ade2-1, can1-100 RAD5, tid1::KanMX* |
| WDHY5359^1^ | *MATα-inc, ura3::LY-HOcs, lys2::LYover0, trp1::GAL-HO hphMX, his3D200 or 11,15, leu2-3,112, ade2-1, can1-100 RAD5, tid1::KanMX* |
| WDHY5355^1^ | *MATa-inc, ura3::LY-HOcs, lys2::LYover0, trp1::GAL-HO hphMX, his3D200 or 11,15, leu2-3,112, ade2-1, can1-100 RAD5, tid1-K352R* |
| WDHY5356^1^ | *MATα-inc, ura3::LY-HOcs, lys2::LYover0, trp1::GAL-HO hphMX, his3D200 or 11,15, leu2-3,112, ade2-1, can1-100 RAD5, tid1-K352R* |
| WDHY4987^1^ | *MATa-/α-inc, ura3::LY-HOcs/-, lys2::LYover0/-, trp1::GAL-HO hphMX/-, his3D200 or 11,15/-, leu2-3,112/-, ade2-1/-, can1-100/- RAD5/-* |
| WDHY5360^1^ | *MATa-/α-inc, ura3::LY-HOcs/-, lys2::LYover0/-, trp1::GAL-HO hphMX/-, his3D200 or 11,15/-, leu2-3,112/-, ade2-1/-, can1-100/- RAD5/-, tid1::KanMX/-* |
| WDHY5370^1^ | *MATa-/α-inc, ura3::LY-HOcs/-, lys2::LYover0/-, trp1::GAL-HO hphMX/-, his3D200 or 11,15/-, leu2-3,112/-, ade2-1/-, can1-100/- RAD5/-, tid1::tid1-K352R/-* |
| WDHY5824^2^ (YAM072)^3^ | *MATa-inc, hoD, ade3::GAL-HO, HMLa-inc, MATa, MATZ2del MATZ1del148bp::Cg-TRP1, hml::MAT::NAT-inc->distal-KAN (148bp homology to MAT), RE-HPHMX, hmr::LEU2, bar1D::ADE3, nej1D::KANMX, ade1, leu2,3-112, trp1::hisG, ura3-52, thr4, lys5* |
| WDHY5825^2^ (YAM075)^3^ | *MATa-inc, hoD, ade3::GAL-HO, HMLa-inc, MATa, hmr::LEU2, bar1D::ADE3, nej1D::KANMX, RE-HPHMX, HMLYαdel::NATMX-inc-2216bpMAT-distal-KANMX, ade1, leu2,3-112, trp1::hisG, ura3-52, thr4, lys5* |
| WDHY5849^2^ | *MATa-inc, hoD, ade3::GAL-HO, HMLa-inc, MATa, MATZ2del MATZ1del148bp::Cg-TRP1, hml::MAT::NAT-inc->distal-KAN (148bp homology to MAT), RE-HPHMX, hmr::LEU2, bar1D::ADE3, nej1D::KANMX, ade1, leu2,3-112, trp1::hisG, ura3-52, thr4, lys5, tid1::URA3* |
| WDHY5850^2^ | *MATa-inc, hoD, ade3::GAL-HO, HMLa-inc, MATa, hmr::LEU2, bar1D::ADE3, nej1D::KANMX, RE-HPHMX, HMLYαdel::NATMX-inc-2216bpMAT-distal-KANMX, ade1, leu2,3-112, trp1::hisG, ura3-52, thr4, lys5, tid1::URA3* |

^1^ W303 strain background.

^2^ S288c strain background.

^3^ Obtained from ([Mehta et al., 2017b](#_ENREF_35)).
